# Supplementary material for: Reproducible diagnostic metabolites in plasma from typhoid fever patients in Asia and Africa
Source: eLife. 2017 May 9;6:e15651. doi: 10.7554/eLife.15651 (PMC5423768; doi:10.7554/eLife.15651)
Supplement: Supplementary file 1. — (A) Table of detected metabolites in plasma samples analyzed with GCxGC-TOFMS in the primary Bangladeshi cohort. (B) Overview of multivariate models. (C) Table of common metabolites between the Bangladeshi and the previous Nepali cohort using OPLS-DA models of culture-positive typhoid infection vs. control. (D) Table of detected metabolites in plasma samples analyzed with GC-TOFMS in the Bangladeshi/Senegalese validation cohort. DOI: http://dx.doi.org/10.7554/eLife.15651.009 [file elife-15651-supp1.docx]

**Supplementary file 1A.** Table of detected metabolites in plasma samples analysed with GCxGC-TOFMS in the primary Bangladeshi cohort.

| **Metabolite^a^** | **ID Info ^b^** | **HMDB ID^c^** | **RT1^d^** | **RT2^d^** | **RI1^d^** | **Significance^e^** | **Direction^f^** |
| --- | --- | --- | --- | --- | --- | --- | --- |
| 1-Monooleoylglycerol | ID |  | 3185 | 2.76 | 2797 | ** # | T |
| 1-Monostearoylglycerol | ID | HMDB31075 | 3180 | 2.81 | 2793 | ** # | T |
| 2,4-Dihydroxybutanoic acid | UC | HMDB00360 | 1245 | 2.96 | 1433 | ** | C |
| 2-Aminobenzoic acid | ID | HMDB01123 | 1835 | 1.87 | 1764 | ** | C |
| 2-Heptanone | ID | HMDB03671 | 505 | 1.22 | 1041 | * | T |
| 2-Hydroxy-3-methylbutyric acid | ID | HMDB00407 | 770 | 2.81 | 1181 | * | T |
| 2-Hydroxypyridine | ID | HMDB13751 | 705 | 1.93 | 1147 | * | T |
| 2-Ketogluconic acid | ID | HMDB11732 | 1615 | 3.46 | 1630 | ** | C |
| 3,4-Dihydroxybutanoic acid | UC | HMDB00337 | 1275 | 2.85 | 1449 | * | C |
| 3-Heptanone | ID | HMDB31482 | 495 | 1.28 | 1035 | (*) | T |
| 3-Hydroxybutyric acid | ID | HMDB00357 | 790 | 2.59 | 1192 | - |  |
| 3-Hydroxyisovaleric acid | ID | HMDB00754 | 865 | 2.53 | 1232 | (*) | T |
| 3-Methyl-2-oxovaleric acid | UC | HMDB00491 | 1035 | 1.99 | 1322 | - |  |
| 4-Hydroxyphenylacetic acid | UC | HMDB00020 | 1850 | 1.91 | 1774 | ** | C |
| 4-Hydroxyproline | ID | HMDB00725 | 1440 | 3.17 | 1537 | ** | T |
| 5-Dodecenoic acid | UC | HMDB00529 | 1405/1405 | 2.12/2.49 | 1518/1518 | ** # | T |
| Alanine | ID | HMDB00161 | 545/545/  1100 | 3.02/3.27/  3.29 | 1062/1062/1356 | - |  |
| Alpha-Ketoisovaleric acid | ID | HMDB00019 | 920 | 1.79 | 1261 | * | T |
| Alpha-Linolenic acid | ID | HMDB01388 | 2650/2650 | 2.04/2.45 | 2335/2335 | ** | T |
| Alpha-Tocopherol | UC | HMDB01893 | 3665 | 2.51 | 3500 | - |  |
| Aminomalonic acid | ID | HMDB01147 | 1440 | 2.61 | 1537 | * | T |
| Arachidonic acid | ID | HMDB01043 | 2855/2860 | 2.32/2.43 | 2512/2516 | - |  |
| Arachidonic acid methyl ester | UC |  | 2815 | 1.79 | 2477 | ** | T |
| Arginine /Citruline /ADMA | UC | HMDB00517/  HMDB00904/  HMDB01539 | 2025 | 2.45 | 1893 | - |  |
| Asparagine | ID | HMDB00168 | 1830/1865 | 2.45/1.82 | 1761/1784 | (*) | T |
| Aspartic acid | ID | HMDB00191 | 1490 | 2.59 | 1563 | - |  |
| Beta-alanine | ID | HMDB00056 | 1250 | 2.98 | 1436 | - |  |
| Campesterol | ID | HMDB02869 | 3755 | 2.82 | 3600 | ** | C |
| Capric acid (Decanoic acid) | ID | HMDB00511 | 1395 | 2.5 | 1513 | ** # | T |
| Caprylic acid (Octanoic acid) | ID | HMDB00482 | 1035/1035 | 2.39/2.54 | 1322/1322 | ** # | T |
| Carbohydrate_248+259 | CL |  | 2160/2230 | 3.79/3.6 | 1985/2033 | * | C |
| Cholesterol | UC | HMDB00067 | 3660 | 2.79 | 3494 | * | T |
| Cholesteryl acetate | UC | HMDB03822 | 3530 | 2.51 | 3151 | - |  |
| Cholic acid | UC | HMDB00619 | 3700 | 2.66 | 3539 | * | C |
| Citric acid | ID | HMDB00094 | 1970 | 2.78 | 1856 | - |  |
| Creatinine | ID | HMDB00562 | 1600/1600 | 2.63/2.64 | 1622/1622 | ** | C |
| Cysteine | ID | HMDB00574 | 1570 | 2.7 | 1606 | ** | C |
| Docosahexaenoic acid | ID | HMDB02183 | 3120 | 2.17 | 2741 | * | T |
| Elaidic acid/Oleic acid | ID | HMDB00573/  HMDB00207 | 2590/2590 | 2.66/2.69 | 2278/2278 | ** # | T |
| Erythritol/Threitol | ID | HMDB02994/  HMDB04136 | 1285/1285 | 4.03/4.13 | 1455/1455 | ** | C |
| Erythrose/Threose | ID | HMDB02649 | 1300/1305 | 2.7/2.74 | 1463/1465 | * | C |
| Ethanolamine | ID | HMDB00149 | 865 | 3.5 | 1232 | * | T |
| Fatty acid_357 | CL |  | 3085 | 2.07 | 2711 | ** | T |
| Fucose | UC | HMDB00174 | 1685 | 3.77 | 1662 | * | T |
| Galacturonic acid | ID | HMDB02545 | 2035 | 3.62 | 1900 | ** | C |
| Gluconic acid | ID | HMDB00625 | 2040 | 4.34 | 1903 | ** | C |
| Glucose or isomer | UC |  | 1895 | 4.23 | 1805 | * | C |
| Glutamic acid | ID | HMDB00148 | 1670 | 2.75 | 1652 | ** | T |
| Glutamine | ID | HMDB00641 | 1550/2005/2005 | 1.94/2.38/  2.44 | 1595/1880/1880 | (*) | T |
| Glutaric acid | UC | HMDB00661 | 1375 | 1.94 | 1502 | * | C |
| Glyceric acid | ID | HMDB06372 | 1095 | 2.7 | 1354 | ** # | T |
| Glycerol-3-phosphate | ID | HMDB00126 | 1905 | 2.75 | 1812 | ** | T |
| Glycine | ID | HMDB00123 | 1015/1015/1010 | 1.28/1.7/  3.25 | 1311/1311/1309 | (*) | T |
| Heptanoic acid | UC | HMDB00666 | 855/855 | 2.25/2.42 | 1226/1226 | * | T |
| Hippuric acid | UC | HMDB00714 | 2445 | 0.92 | 2179 | * | C |
| Hydroxylamine | UC | HMDB03338 | 600 | 3.24 | 1091 | (*) | C |
| Hydroxyphenyllactic acid | UC | HMDB00755 | 2155 | 2.55 | 1982 | ** | C |
| Indoleacetic acid | ID | HMDB00197 | 2510 | 1.8 | 2223 | (*) | T |
| Inosine | UC | HMDB00195 | 3110 | 2.11 | 2733 | ** # | C |
| Isoleucine | UC | HMDB00172 | 935/1000 | 1.9/3.04 | 1269/1303 | * | T |
| Ketoleucine | ID | HMDB00695 | 1060 | 1.95 | 1335 | (*) | C |
| Lactic acid | ID | HMDB00190 | 615 | 2.43 | 1099 | ** | T |
| Lactose | ID | HMDB00186 | 2860 | 4.71 | 2516 | ** | C |
| Leucine | ID | HMDB00687 | 960 | 3.01 | 1282 | * | T |
| Linoleic acid (9,12-Octadecadienoic acid) | ID | HMDB00673 | 2615/2615 | 2.49/2.56 | 2304/2304 | ** | T |
| Lysine | ID | HMDB00182 | 2010/2035 | 3.57/2.91 | 1883/1900 | (*) | C |
| m-Cresol | UC | HMDB02048 | 905 | 2.2 | 1253 | ** | C |
| Methionine | ID | HMDB00696 | 1550/1570/1570 | 1.76/2.38/  2.5 | 1595/1606/1606 | - |  |
| Methyl hexadecanoic acid | ID | HMDB61859 | 2255 | 2.22 | 2050 | ** # | T |
| Monoethylhexyl phthalic acid | UC | HMDB13248 | 3220 | 1.79 | 2827 | * | T |
| Monomethylphosphate? | UC |  | 1000 | 2.05 | 1303 | ** | T |
| Monosaccharide_203+209 | CL |  | 1890/1915 | 4.12/4.03 | 1801/1812 | * | C |
| Monosaccharide_231+232 | CL |  | 2060/2060 | 3.55/4.04 | 1917/1917 | (*) | T |
| Myo-inositol | ID | HMDB00211 | 2130 | 4.49 | 1965 | ** | C |
| Myo-inositol-1-phosphate | ID | HMDB00213 | 2630/2630 | 4.05/4.09 | 2317/2317 | ** # | T |
| Myo-inositol-2-phosphate | UC |  | 2715 | 3.72 | 2391 | ** # | T |
| Nonanoic acid | ID | HMDB00847 | 1220 | 2.61 | 1420 | ** # | T |
| Ornithine | ID | HMDB00214 | 1850/1875 | 3.68/2.92 | 1774/1791 | - |  |
| Palmitic acid (Hexadecanoic acid) | ID | HMDB00220 | 2330 | 2.74 | 2101 | ** # | T |
| Palmitoleic acid (Hexadecenoic acid) | ID | HMDB03229 | 2335 | 2.54 | 2104 | ** | T |
| Pentitol (ribitol), 3-desoxy | UC |  | 1480 | 3.87 | 1558 | ** # | T |
| Phenylalanine | ID | HMDB00159 | 1770/1770/1815 | 2.06/2.49/  1.76 | 1720/1720/1750 | (*) | T |
| Phenyllactic acid | UC | HMDB00779 | 1710 | 2.42 | 1679 | (*) | C |
| Phosphoric acid | ID | HMDB02142 | 1070/1017 | 2.35/2.5 | 1341/1341 | * | T |
| Proline | UC | HMDB00162 | 1085 | 2.77 | 1348 | * | T |
| Pseudouridine | UC | HMDB00767 | 2700 | 2.82 | 2378 | * | C |
| Pyroglutamic acid | UC | HMDB00267 | 1705 | 2.13 | 1676 | - |  |
| Pyruvic acid | ID | HMDB00243 | 770/795 | 1.76/1.46 | 1181/1195 | ** | C |
| Saccharide_182 | CL |  | 1790 | 4.14 | 1733 | * # | C |
| Serine | ID | HMDB00187 | 1060/1060/1130 | 2.2/2.44/  3.09 | 1335/1335/1346 | ** | T |
| S-methylcysteine | UC | HMDB02108 | 1390 | 2.46 | 1510 | * | T |
| Stearic acid (Octadecanoic acid) | ID | HMDB00827 | 2590 | 2.79 | 2278 | ** | T |
| Sucrose | ID | HMDB00258 | 2825 | 4.52 | 2486 | - |  |
| Sugar alcohol_151 | CL |  | 1600 | 4.66 | 1622 | * | C |
| Sugar alcohol_154 | CL |  | 1610 | 4.5 | 1627 | ** | C |
| Sugar alcohol_237 | CL |  | 2100 | 3.63 | 1944 | * | C |
| Threonic/Erytrheonic acid | ID | HMDB00943/  HMDB00613 | 1425/1480/1480 | 3.41/3.09/  3.29 | 1529/1558/1558 | * | C |
| Threonine | ID | HMDB00167 | 1100/1155/1155 | 2.53/3.13/  3.32 | 1356/1386/1386 | - |  |
| Tyrosine | ID | HMDB00158 | 2200 | 2.53 | 2012 | (*) | C |
| Uncertain_041 | UC | HMDB00666 | 905 | 2.29 | 1253 | - |  |
| Uncertain_195 | UC |  | 1855 | 1.8 | 1778 | (*) | T |
| Unknown_004 | UN |  | 535 | 1.93 | 1057 | ** | T |
| Unknown_007 | UN |  | 555 | 2.14 | 1067 | - |  |
| Unknown_008 | UN |  | 560 | 4.09 | 1070 | * | C |
| Unknown_009 | UN |  | 575 | 1.88 | 1078 | ** | T |
| Unknown_010 | UN |  | 580 | 3.3 | 1081 | * | C |
| Unknown_019 | UN |  | 740 | 2.42 | 1165 | * | T |
| Unknown_020 | UN |  | 740 | 2.48 | 1165 | ** | T |
| Unknown_021 | UN |  | 745 | 2.61 | 1168 | * | C |
| Unknown_022 | UN |  | 750 | 2.52 | 1171 | - |  |
| Unknown_028 | UN |  | 795 | 2.65 | 1195 | ** | C |
| Unknown_047 | UN |  | 975 | 2.72 | 1290 | * | T |
| Unknown_050 | UN |  | 1000 | 3 | 1303 | ** | T |
| Unknown_055 | UN |  | 1045 | 1.64 | 1327 | - |  |
| Unknown_062 | UN |  | 1075 | 0.94 | 1343 | * | T |
| Unknown_065+066 | UN |  | 1075/1075 | 3.61/4.1 | 1343/1343 | (*) | C |
| Unknown_080+082 | UN |  | 1175/1190 | 1.64/1.57 | 1396/1404 | ** | C |
| Unknown_084 | UN |  | 1200 | 1.97 | 1409 | * | C |
| Unknown_090 | UN |  | 1225 | 3.8 | 1423 | ** | T |
| Unknown_091 | UN |  | 1250 | 1.94 | 1436 | * | T |
| Unknown_094 | UN |  | 1265 | 0.21 | 1444 | * | T |
| Unknown_095 | UN |  | 1260 | 1.74 | 1441 | - |  |
| Unknown_103 | UN |  | 1300 | 3.63 | 1463 | ** | T |
| Unknown_105 | UN |  | 1305 | 2.33 | 1465 | ** | C |
| Unknown_107 | UN |  | 1325 | 2.6 | 1476 | - |  |
| Unknown_108 | UN |  | 1330 | 2.64 | 1478 | ** # | C |
| Unknown_109 | UN |  | 1340 | 2.51 | 1484 | * | T |
| Unknown_110+111 | UN |  | 1360/1360 | 1.38/1.63 | 1494/1494 | * | T |
| Unknown_112 | UN |  | 1360 | 1.9 | 1494 | * | T |
| Unknown_114 | UN |  | 1365 | 2.93 | 1497 | - |  |
| Unknown_116 | UN |  | 1390 | 3.45 | 1510 | - |  |
| Unknown_121 | UN |  | 1410 | 2.69 | 1521 | - |  |
| Unknown_122 | UN |  | 1425 | 3.38 | 1529 | ** | C |
| Unknown_125 | UN |  | 1440 | 2.62 | 1537 | ** | T |
| Unknown_127 | UN |  | 1475 | 1.9 | 1555 | * | T |
| Unknown_132 | UN |  | 1505 | 1.97 | 1571 | ** | T |
| Unknown_133 | UN |  | 1510 | 3.29 | 1574 | * | C |
| Unknown_134 | UN |  | 1530 | 0.97 | 1585 | * | T |
| Unknown_136 | UN |  | 1535 | 2.69 | 1587 | ** | T |
| Unknown_142 | UN |  | 1565 | 2.04 | 1603 | * | T |
| Unknown_147 | UN |  | 1580 | 1.32 | 1611 | ** | T |
| Unknown_148 | UN |  | 1595 | 2.48 | 1619 | - |  |
| Unknown_157+164 | UN |  | 1660/1600 | 0.04/0.03 | 1645/1658 | * | C |
| Unknown_159 | UN |  | 1655 | 2.77 | 1641 | * | C |
| Unknown_161 | UN |  | 1670 | 1.64 | 1652 | * | C |
| Unknown_165 | UN |  | 1685 | 2.44 | 1662 | * | C |
| Unknown_169 | UN |  | 1700 | 3.36 | 1672 | * | C |
| Unknown_170 | UN |  | 1695 | 3.51 | 1669 | * | T |
| Unknown_172 | UN |  | 1725 | 4.15 | 1689 | * | C |
| Unknown_173 | UN |  | 1745 | 2.77 | 1703 | * | T |
| Unknown_179 | UN |  | 1780 | 2.5 | 1727 | * | C |
| Unknown_180 | UN |  | 1775 | 2.51 | 1723 | * | T |
| Unknown_183 | UN |  | 1810 | 2.03 | 1747 | - |  |
| Unknown_185 | UN |  | 1815 | 0.87 | 1750 | * | C |
| Unknown_188 | UN |  | 1820 | 3.52 | 1754 | ** | C |
| Unknown_189+190 | UN |  | 1840/1840 | 1.56/1.67 | 1767/1767 | (*) | T |
| Unknown_193 | UN |  | 1835 | 2.67 | 1764 | * | C |
| Unknown_200 | UN |  | 1870 | 2.43 | 1788 | - |  |
| Unknown_201 | UN |  | 1885 | 2.49 | 1798 | ** | C |
| Unknown_205 | UN |  | 1895 | 4.25 | 1805 | ** # | C |
| Unknown_207+210 | UN |  | 1910/1930 | 1.62/1.63 | 1815/1829 | * | C |
| Unknown_211+217 | UN |  | 1925/1950 | 1.75/1.95 | 1825/1842 | ** | C |
| Unknown_213 | UN |  | 1930 | 2.65 | 1829 | ** | C |
| Unknown_214 | UN |  | 1930 | 2.96 | 1829 | - |  |
| Unknown_215 | UN |  | 1940 | 1.67 | 1835 | * | C |
| Unknown_216 | UN |  | 1945 | 1.87 | 1839 | * | T |
| Unknown_219 | UN |  | 1990 | 3.44 | 1869 | * | T |
| Unknown_220 | UN |  | 2000 | 1.7 | 1876 | ** | T |
| Unknown_223 | UN |  | 2020 | 1.81 | 1890 | * | C |
| Unknown_228 | UN |  | 2050 | 1.71 | 1910 | * | T |
| Unknown_233 | UN |  | 2070 | 3.33 | 1924 | * | C |
| Unknown_238 | UN |  | 2105 | 4.05 | 1948 | * | C |
| Unknown_242 | UN |  | 2135 | 1.92 | 1968 | * | T |
| Unknown_246+253 | UN |  | 2150/2190 | 3.08/3 | 1978/2005 | * | C |
| Unknown_249 | UN |  | 2180 | 2.01 | 1999 | ** | T |
| Unknown_251 | UN |  | 2190 | 2.18 | 2005 | * | C |
| Unknown_257+260 | UN |  | 2230/2235 | 1.83/1.88 | 2033/2036 | ** | C |
| Unknown_262 | UN |  | 2260 | 3.46 | 2053 | ** | C |
| Unknown_265 | UN |  | 2300 | 1.68 | 2080 | ** | T |
| Unknown_266 | UN |  | 2320 | 1.72 | 2094 | ** # | C |
| Unknown_270 | UN |  | 2350 | 3.57 | 2114 | ** | C |
| Unknown_271 | UN |  | 2370 | 2.8 | 2128 | ** | C |
| Unknown_272 | UN |  | 2385 | 2.09 | 2138 | ** | C |
| Unknown_273 | UN |  | 2420 | 1.74 | 2162 | * | C |
| Unknown_277 | UN |  | 2450 | 2.19 | 2182 | * | T |
| Unknown_278 | UN |  | 2450 | 3.35 | 2182 | ** | C |
| Unknown_279+282 | UN |  | 2450/2475 | 3.66/3.66 | 2182/2199 | * | C |
| Unknown_280 | UN |  | 2465 | 4.43 | 2193 | ** | C |
| Unknown_281+283 | UN |  | 2475/2495 | 0.19/0.17 | 2199/2213 | * | T |
| Unknown_284 | UN |  | 2490 | 1.62 | 2210 | ** | C |
| Unknown_287+295 | UN |  | 2515/2550 | 3.63/3.63 | 2227/2250 | * | C |
| Unknown_291 | UN |  | 2520 | 3.59 | 2230 | * | C |
| Unknown_292 | UN |  | 2535 | 2.94 | 2240 | ** # | T |
| Unknown_293 | UN |  | 2550 | 0.82 | 2250 | (*) | T |
| Unknown_294 | UN |  | 2555 | 2.19 | 2254 | ** # | T |
| Unknown_296+301 | UN |  | 2575/2595 | 3.6/3.65 | 2267/2281 | ** | C |
| Unknown_300 | UN |  | 2585 | 3.47 | 2274 | ** | C |
| Unknown_308 | UN |  | 2660 | 1.87 | 2343 | ** | C |
| Unknown_310 | UN |  | 2655 | 3.31 | 2339 | ** | C |
| Unknown_311 | UN |  | 2680 | 1.84 | 2361 | * | T |
| Unknown_313+318 | UN |  | 2700/2700 | 2.43/2.42 | 2378/2378 | ** | T |
| Unknown_314 | UN |  | 2690 | 2.48 | 2369 | * | T |
| Unknown_320 | UN |  | 2710 | 4.14 | 2387 | * | C |
| Unknown_323 | UN |  | 2760 | 1.74 | 2430 | * | T |
| Unknown_328+331+332 | UN |  | 2805/2825/2825 | 3.92/3.87/  4.07 | 2469/2486/2486 | ** | C |
| Unknown_330 | UN |  | 2825 | 2.6 | 2486 | ** | T |
| Unknown_335+341 | UN |  | 2860/2865 | 1.99/2.02 | 2516/2521 | * | T |
| Unknown_338 | UN |  | 2855 | 2.86 | 2512 | ** | C |
| Unknown_344+346 | UN |  | 2905/2910 | 1.81/1.89 | 2555/2560 | ** | T |
| Unknown_348+350 | UN |  | 2930/2930 | 2.18/2.46 | 2577/2577 | - |  |
| Unknown_349 | UN |  | 2930 | 2.42 | 2577 | * | T |
| Unknown_351 | UN |  | 2935 | 2.5 | 2581 | - |  |
| Unknown_352 | UN |  | 2945 | 1.97 | 2590 | * | T |
| Unknown_353 | UN |  | 2960 | 2.2 | 2603 | ** | C |
| Unknown_354 | UN |  | 3030 | 3.27 | 2663 | - |  |
| Unknown_355 | UN |  | 3045 | 1.65 | 2676 | (*) | T |
| Unknown_356 | UN |  | 3075 | 1.63 | 2702 | * | C |
| Unknown_358 | UN |  | 3085 | 2.59 | 2711 | * | C |
| Unknown_359 | UN |  | 3090 | 2.59 | 2715 | ** | C |
| Unknown_361 | UN |  | 3110 | 2.51 | 2733 | ** # | C |
| Unknown_363+364 | UN |  | 3140/3140 | 1.97/2.49 | 2759/2759 | * | T |
| Unknown_365+366 | UN |  | 3165/3190 | 2.43/2.42 | 2780/2802 | ** | C |
| Unknown_367 | UN |  | 3180 | 2.56 | 2793 | ** | C |
| Unknown_371 | UN |  | 3275 | 3.22 | 2885 | * | C |
| Unknown_372 | UN |  | 3400 | 1.65 | 3015 | ** | C |
| Unknown_374 | UN |  | 3440 | 2.02 | 3057 | ** # | C |
| Unknown_377 | UN |  | 3525 | 2.45 | 3146 | ** | T |
| Unknown_379 | UN |  | 3575 | 1.22 | 3198 | * | C |
| Unknown_380 | UN |  | 3645 | 1.82 | 3478 | (*) | T |
| Unknown_383 | UN |  | 3670 | 0.16 | 3506 | (*) | C |
| Unknown_387 | UN |  | 3725 | 2.77 | 3567 | ** # | T |
| Unknown_393+394 | UN |  | 3950/3960 | 3043/3.41 | 3816/3827 | * | T |
| Urea | ID | HMDB00294 | 1155 | 2.02 | 1386 | * | C |
| Valine | ID | HMDB00883 | 765/850 | 2.1/2.71 | 1179/1224 | * | T |
| Xylobiose | ID | HMDB29894 | 2860 | 4.11 | 2516 | ** # | C |
| Xylulose | ID | HMDB01644 | 1660 | 3.5 | 1645 | * | C |

^a^Metabolites: Metabolite name where name within parenthesis refers to another synonym and name after the slash refers to another isomer or other cases where complete distinction between the metabolites is not possible with the used technique.

^b^ID Info refers to level of identification; ID: putatively annotated metabolite, CL: assigned metabolite class, UC: uncertain identity, UN: unknown identity

^c^HMDB ID; ID number from the Human Metabolome Database

^d^RT1 refers to the 1^st^ dimension retention time (s), RT2 refers to the 2^nd^ dimension retention time (s), RI1 refers to the 1^st^ dimension retention index. Multiple entries refers to individual values included in summed peak.

^e^Significance refers to multivariate and univariate significance criteria where metabolites significant multivariate with w* > | ± SD| are marked with **, metabolites significant multivariate with w* > |0.03| are marked with *, metabolites borderline significant multivariate are marked with (*), metabolites that are significant univariate with p ≤ 0.05 are marked with # and metabolites that are not significant are marked with “-“.

^f^ Direction refers to direction of change in relative metabolite concentration in OPLS-DA models comparing patients with culture positive typhoid infection and patients from a control group, where metabolites with higher relative concentration in the control group are marked with C and metabolites with higher relative concentration in the typhoid group are marked with T.

**Supplementary file 1B.** Overview of multivariate models

| Model^a^ | Cohort^b^ | Biofluid | Method^c^ | Num. met^d^ | Comp.^e^ | R^2^X^f^ | R^2^Y^f^ | Q^2f^ | CV-ANOVA^g^ |
| --- | --- | --- | --- | --- | --- | --- | --- | --- | --- |
| Typhoid vs. febrile control | Bangladeshi | Plasma | GCxGC | 236 | 1+1 | 0.31 | 0.804 | 0.598 | 5.9*10^-3^ |
| Typhoid vs. febrile control | Bangladeshi | Plasma | GCxGC | 33 | 1+1 | 0.52 | 0.659 | 0.411 | 7.7*10^-2^ |
| Typhoid vs. afebrile control | Nepali | Plasma | GCxGC | 33 | 1+1 | 0.362 | 0.837 | 0.747 | 3.0*10^-17^ |
| Typhoid vs. febrile control | Bangladeshi | Plasma | GCxGC | 15 | 1+1 | 0.586 | 0.688 | 0.535 | 1.6*10^-2^ |
| Typhoid vs. afebrile control | Nepali | Plasma | GCxGC | 15 | 1+1 | 0.423 | 0.792 | 0.722 | 4.8*10^-16^ |
| Typhoid vs febrile control (pos mode) | Bangladeshi | Urine | LC | 941 | 1+1 | 0.375 | 0.857 | 0.503 | 2.5*10^-2^ |
| Typhoid vs febrile control (neg mode) | Bangladeshi | Urine | LC | 754 | 1+1 | 0.36 | 0.828 | 0.5 | 2.0*10^-2^ |
| Typhoid vs malaria vs other controls (bacteria/pathogen) (3 classes) | Bangladeshi/ Senegalese | Plasma | GC | 104 | 2+2 | 0.555 | 0.599 | 0.369 | 3.5*10^-3^ |
| Typhoid vs control (malaria + other bacteria/pathogen) | Bangladeshi/ Senegalese | Plasma | GC | 104 | 1+1 | 0.442 | 0.609 | 0.404 | 7.2*10^-5^ |
| Typhoid vs malaria | Bangladeshi/ Senegalese | Plasma | GC | 104 | 1+1 | 0.491 | 0.745 | 0.613 | 1.3*10^-4^ |
| Typhoid vs control (malaria + other bacteria/pathogen) | Bangladeshi/Senegalese | Plasma | GC | 24 | 1+1 | 0.565 | 0.518 | 0.406 | 3.1*10^-5^ |
| Typhoid vs. febrile control | Bangladeshi | Plasma | GCxGC | 13 | 1+1 | 0.638 | 0.471 | 0.227 | 3.9*10^-1^ |
| Typhoid vs. afebrile control | Nepali | Plasma | GCxGC | 14 | 1+1 | 0.482 | 0.733 | 0.598 | 2.5*10^-11^ |

^a^All models are two-class OPLS-DA models unless stated otherwise.

^b^Cohort; Bangladeshi: current study, Nepali: previous study, Bangladeshi/Senegalese: validation cohort.

^c^Method: Analytical method used; GCxGC: GCxGC-TOFMS, LC: UHPLC-Q-TOFMS, GC: GC-TOFMS.

^d^Num. met: The number of metabolites the model is based on.

^e^Comp: The number of predictive model components followed by the number of orthogonal model components.

^f^R^2^X: The amount of variation in X explained by the model, R^2^Y: The amount of variation in Y explained by the model, Q^2^: The amount of variation in Y predicted by the model.

^g^p CV-ANOVA: p-value based on cross-validated scores showing the degree of significance for the separation.

**Supplementary file 1C.** Table of metabolites in common between the Bangladeshi and previous Nepali cohorts using OPLS-DA models of culture positive typhoid infection vs. control

| **Metabolite^a^** | **ID Info^b^** | **HMDB ID^c^** | **Significance TY2^d^** | **Direction TY2^e^** | **Direction TY1^e^** |
| --- | --- | --- | --- | --- | --- |
| **Consistently up or down regulated in TY2 and TY1** | | | | | |
| 1-Monostearoylglycerol | ID | HMDB31075 | ** # | T | T |
| 2-Hydroxy-3-methylbutyric acid | ID | HMDB00407 | * | T | T |
| 3-Hydroxyisovaleric acid | ID | HMDB00754 | (*) | T | T |
| 5-Dodecenoic acid | UC | HMDB00529 | ** # | T | T |
| Asparagine | ID | HMDB00168 | (*) | T | T |
| Capric acid (Decanoic acid) | ID | HMDB00511 | ** # | T | T |
| Caprylic acid (Octanoic acid) | ID | HMDB00482 | ** # | T | T |
| Docosahexaenoic acid | ID | HMDB02183 | * | T | T |
| Elaidic acid/Oleic acid | ID | HMDB00573/HMDB00207 | ** # | T | T |
| Fucose | UC | HMDB00174 | * | T | T |
| Galacturonic acid | ID | HMDB02545 | ** | C | C |
| Glutamine | ID | HMDB00641 | (*) | T | T |
| Glyceric acid | ID | HMDB06372 | ** # | T | T |
| Glycerol-3-phosphate | ID | HMDB00126 | ** | T | T |
| Leucine | ID | HMDB00687 | * | T | T |
| Linoleic acid (9,12-Octadecadienoic acid) | ID | HMDB00673 | ** | T | T |
| Lysine | ID | HMDB00182 | (*) | C | C |
| Monosaccharide_231+232 | CL |  | (*) | T | T |
| Palmitoleic acid (Hexadecenoic acid) | ID | HMDB03229 | ** | T | T |
| Pentitol (ribitol), 3-desoxy | UC |  | ** # | T | T |
| Phenylalanine | ID | HMDB00159 | (*) | T | T |
| Stearic acid (Octadecanoic acid) | ID | HMDB00827 | ** | T | T |
| Sugar alcohol_237 | CL |  | * | C | C |
| Tyrosine | ID | HMDB00158 | (*) | C | C |
| Unknown_219 | UN |  | * | T | T |
| Unknown_246+253 | UN |  | * | C | C |
| Unknown_279+282 | UN |  | * | C | C |
| Unknown_280 | UN |  | ** | C | C |
| Unknown_287+295 | UN |  | * | C | C |
| Unknown_335+341 | UN |  | * | T | T |
| Unknown_338 | UN |  | ** | C | C |
| Unknown_344+346 | UN |  | ** | T | T |
| Unknown_363+364 | UN |  | * | T | T |
| **Inconsistently up or down regulated in TY2 and TY1** | | | | | |
| 2,4-Dihydroxybutanoic acid | UC | HMDB00360 | ** | C | T |
| 3,4-Dihydroxybutanoic acid | UC | HMDB00337 | * | C | T |
| Aminomalonic acid | ID | HMDB01147 | * | T | C |
| Campesterol | ID | HMDB02869 | ** | C | T |
| Cholesterol | UC | HMDB00067 | * | T | C |
| **Inconsistently up or down regulated in TY2 and TY1** | | | | | |
| Creatinine | ID | HMDB00562 | ** | C | T |
| Cysteine | ID | HMDB00574 | ** | C | T |
| Erythritol/Threitol | ID | HMDB02994/HMDB04136 | ** | C | T |
| Gluconic acid | ID | HMDB00625 | ** | C | T |
| Hydroxyphenyllactic acid | UC | HMDB00755 | ** | C | T |
| Ketoleucine | ID | HMDB00695 | (*) | C | T |
| Methyl hexadecanoic acid | ID | HMDB61859 | ** # | T | C |
| Myo-inositol-1-phosphate | ID | HMDB00213 | ** # | T | C |
| Phenyllactic acid | UC | HMDB00779 | (*) | C | T |
| Pseudouridine | UC | HMDB00767 | * | C | T |
| Pyruvic acid | ID | HMDB00243 | ** | C | T |
| Saccharide_182 | CL |  | * # | C | T |
| S-methylcysteine | UC | HMDB02108 | * | T | C |
| Unknown_021 | UN |  | * | C | T |
| Unknown_090 | UN |  | ** | T | C |
| Unknown_292 | UN |  | ** # | T | C |
| Unknown_080+082 | UN |  | ** | C | T |
| Urea | ID | HMDB00294 | * | C | T |

TY1=previous study, Nepali cohort, TY2=current study, Bangladeshi cohort

^a^Metabolite: Metabolite name where name within parenthesis refers to another synonym and name after the slash refers to another isomer or other cases where complete distinction between the metabolites is not possible with the used technique.

^b^ID Info refers to level of identification; ID: putatively annotated metabolite, CL: assigned metabolite class, UC: uncertain identity, UN: unknown identity

^c^HMDB ID; ID number from the Human Metabolome Database

^d^Significance TY2 refers to multivariate and univariate significance criteria where metabolites significant multivariate with w* > | ± SD| are marked with **, metabolites significant multivariate with w* > |0.03| are marked with *, metabolites borderline significant multivariate are marked with (*) and metabolites that are significant univariate with p ≤ 0.05 are marked with #. Regarding significance in TY1 all metabolites are multivariate (w* > |0.03|) and univariate (p ≤ 0.05) significant.

^e^Direction refers to direction of change in relative metabolite concentration in OPLS-DA models comparing patients with culture positive typhoid infection and patients from a control group, where metabolites with higher relative concentration in the control group are marked with C and metabolites with higher relative concentration in the typhoid group are marked with T.

**Supplementary file 1D.** Table of detected metabolites in plasma samples analysed with GC-TOFMS in the Bangladeshi/Senegalese validation cohort

| **Metabolite^a^** | **ID Info^b^** | **HMDB ID^c^** | **RI^d^** | **Significance^e^** | **Direction^f^** | **Consistantly regulated^g^** |
| --- | --- | --- | --- | --- | --- | --- |
| 1,5-anhydro-D-glucitol | ID | HMDB02712 | 1846 | - |  |  |
| 1-Palmitoyl-glycero-3-phosphocholine | UC | HMDB10382 | 3031 | **# | C |  |
| 2-aminobutyric acid | ID | HMDB00452 | 1187 | *# | T | TY1 |
| 2-methylmalic acid | ID | HMDB00426 | 1467 | * | T |  |
| 3-hydroxybutyric acid | ID | HMDB00357 | 1178 | * | C |  |
| 4-aminobutyric acid | ID | HMDB00112 | 1527 | *# | T |  |
| Aconitic acid | ID | HMDB00072 | 1742 | **# | T |  |
| Adenosine-5-monophosphate | ID | HMDB00045 | 3057 | - |  |  |
| Adipic acid | ID | HMDB00448 | 1502 | * | T |  |
| Alanine | ID | HMDB00161 | 1127 | * | T |  |
| Alpha-ketoglutaric acid | ID | HMDB00208 | 1571 | **# | T |  |
| Arachidonic acid | ID | HMDB01043 | 2364 | - |  |  |
| Benzoic acid | UC | HMDB01870 | 1256 | **# | C |  |
| Beta-Alanine | ID | HMDB00056 | 1425 | **# | T | TY1 |
| Carbohydrate_070 | UC |  | 1888 | - |  |  |
| Cholesterol | ID | HMDB00067 | 3162 | - |  |  |
| Citric acid | ID | HMDB00094 | 1812 | - |  |  |
| Citrulline (Ornithine) | UC | HMDB00904 | 1613 | * | T |  |
| Creatinine | UC | HMDB00562 | 1549 | **# | T | TY1 |
| Cystine | ID | HMDB00192 | 2286 | *# | T |  |
| Cytosine | ID | HMDB00630 | 1540 | **# | T |  |
| Docosahexaenoic acid | ID | HMDB02183 | 2553 | - |  |  |
| Docosapentaenoic acid | UC | HMDB01976 | 2554 | * | C |  |
| Dodecanoic acid | ID | HMDB00638 | 1650 | - |  |  |
| Elaidic/Oleic acid | ID | HMDB00573/HMDB00207 | 2216 | - |  |  |
| Erythritol/Threitol | ID | HMDB02994/HMDB04136 | 1502 | **# | T | TY1 |
| Ethanolamine | ID | HMDB00149 | 1272 | **# | T | TY2 |
| Fructose | UC | HMDB00660 | 1867 | **# | C | TY2 |
| Fructose-6-Phosphate | UC | HMDB00124 | 2295 | *# | T |  |
| Fumaric acid | ID | HMDB00134 | 1352 | **# | T |  |
| Galacturonic acid | UC | HMDB02545 | 1930 | - |  |  |
| Gluconic acid 1,5-lactone | UC | HMDB00150 | 1868 | *# | T |  |
| Glucose | UC | HMDB00122 | 1881 | **# | C | TY2 |
| Glucose-6-phospate | ID | HMDB01401 | 2308 | * | T |  |
| Glutamic acid | ID | HMDB00148 | 1618 | * | C |  |
| Glutaric acid | ID | HMDB00661 | 1403 | **# | T |  |
| Glyceric acid | ID | HMDB06372 | 1332 | - |  |  |
| Glycerol | ID | HMDB00131 | 1266 | * | C |  |
| Glycerol-2-phosphate | ID | HMDB02520 | 1717 | - |  |  |
| Glycerol-3-phosphate | ID | HMDB00126 | 1755 | *# | T | TY2/TY1 |
| Glycine | ID | HMDB00123 | 1308 | * | T | TY2 |
| Glycolic acid | ID | HMDB00115 | 1104 | **# | C |  |
| Hydroxyproline | ID | HMDB00725 | 1521 | **# | C |  |
| Hypoxanthine | ID | HMDB00157 | 1800 | * | T | TY1 |
| Inosine | ID | HMDB00195 | 2564 | * | C | TY2 |
| Isoleucine | ID | HMDB00172 | 1298 | * | T | TY2 |
| Isomaltose | ID | HMDB02923 | 2862 | * | C |  |
| Lactose | ID | HMDB00186 | 2680 | - |  |  |
| Leucine | ID | HMDB00687 | 1279 | * | T | TY2/TY1 |
| Levulinic acid | UC | HMDB00720 | 1254 | **# | T |  |
| Linoleic acid (9,12-Octadecadienoic acid) | ID | HMDB00673 | 2208 | * | C |  |
| Lysine | ID | HMDB00182 | 1918 | - |  |  |
| Malic acid | ID | HMDB00744 | 1484 | * | T | TY1 |
| Maltose | ID | HMDB00163 | 2728 | - |  |  |
| Mannitol | ID | HMDB00765 | 2131 | **# | T |  |
| Methionine | ID | HMDB00696 | 1515 | **# | T |  |
| Monosaccharide_067 | UC |  | 2111 | **# | C |  |
| Monosaccharide_186 | UC |  | 1875 | *# | C | TY2 |
| Monosaccharide_188 | UC |  | 1710 | *# | T |  |
| Monosaccharide_190 | UC |  | 1898 | **# | C | TY2 |
| Monosaccharide_247 | UC |  | 1676 | **# | T |  |
| Myo-Inositol | ID | HMDB00211 | 2084 | - |  |  |
| Myristoleic acid | ID | HMDB02000 | 1832 | * | C |  |
| O-Phosphoethanolamine | ID | HMDB00224 | 1776 | * | T |  |
| Ornithine | ID | HMDB00214 | 1811 | - |  |  |
| Palmitic acid (Hexadecanoid acid) | ID | HMDB00220 | 2046 | - |  |  |
| Pantothenic acid | UC | HMDB00210 | 1985 | *# | T |  |
| Phenylalanine | ID | HMDB00159 | 1623 | * | T | TY2/TY1 |
| Pipecolic acid | ID | HMDB00070 | 1366 | **# | T | TY1 |
| Pyroglutamic acid | ID | HMDB00267 | 1519 | **# | C |  |
| Pyruvic acid | UC | HMDB00243 | 1074 | * | C | TY2 |
| Ribitol (or isomer) | UC | HMDB00508 | 1717 | * | T |  |
| Ribose (or isomer) | UC | HMDB00283 | 1655 | **# | T |  |
| Saccharide_219 | UC |  | 2755 | **# | C |  |
| Sorbitol | UC | HMDB00247 | 1927 | * | C |  |
| Stearic acid (Octadecanoic acid) | ID | HMDB00827 | 2241 | **# | T | TY2/TY1 |
| Succinic acid | ID | HMDB00254 | 1319 | * | T |  |
| Sucrose | ID | HMDB00258 | 2631 | * | C |  |
| Sugar alcohol_069 | UC |  | 2271 | **# | C |  |
| Sugar alcohol_125 | UC |  | 2083 | **# | C |  |
| Sugar alcohol_127 | UC |  | 2070 | *# | T |  |
| Sugar alcohol_192+193 | UC |  | 1915/ 1913 | **# | C |  |
| Taurine | ID | HMDB00251 | 1662 | - |  |  |
| Threonine | ID | HMDB00167 | 1384 | - |  |  |
| Thymine | ID | HMDB00262 | 1397 | **# | T |  |
| Trehalose | ID | HMDB00975 | 2736 | * | T |  |
| Tryptophan | ID | HMDB00929 | 2207 | *# | C | TY1 |
| Tyrosine | ID | HMDB00158 | 1935 | * | T |  |
| Unknown_018 | UN |  | 2773 | *# | T |  |
| Unknown_019 | UN |  | 2539 | *# | T |  |
| Unknown_037 | UN |  | 1855 | *# | T |  |
| Unknown_038 | UN |  | 1858 | **# | C |  |
| Unknown_045 | UN |  | 1551 | **# | T |  |
| Unknown_066 | UN |  | 1950 | *# | C | TY2/TY1 |
| Unknown_143 | UN |  | 1261 | **# | T |  |
| Unknown_156 | UN |  | 1792 | **# | T |  |
| Unknown_160 | UN |  | 2458 | - |  |  |
| Unknown_184 | UN |  | 1937 | - |  |  |
| Unknown_206 | UN |  | 1969 | **# | C |  |
| Uracil | ID | HMDB00300 | 1339 | **# | T | TY1 |
| Urea | ID | HMDB00294 | 1191 | - |  |  |
| Uric acid | ID | HMDB00289 | 2093 | * | C |  |
| Valine | ID | HMDB00883 | 1230 | * | T | TY2 |
| Xylose (or isomer) | UC | HMDB00098 | 1672 | **# | T |  |

^a^Metabolites: Metabolite name where name within parenthesis refers to another synonym and name after the slash refers to another isomer or other cases where complete distinction between the metabolites is not possible with the used technique.

^b^ID Info refers to level of identification; ID: putatively annotated metabolite, CL: assigned metabolite class, UC: uncertain identity, UN: unknown identity

^c^HMDB ID; ID number from the Human Metabolome Database

^d^RI1 refers to the 1^st^ dimension retention index. Multiple entries refers to individual values included in summed peak.

^e^Significance refers to multivariate and univariate significance criteria where metabolites significant multivariate with w* > | ± SD| are marked with **, metabolites significant multivariate with w* > |0.03| are marked with *, metabolites borderline significant multivariate are marked with (*), metabolites that are significant univariate with p ≤ 0.05 are marked with # and metabolites that are not significant are marked with “-“.

^f^ Direction refers to direction of change in relative metabolite concentration in OPLS-DA models comparing patients with culture positive typhoid infection and patients from a control group, where metabolites with higher relative concentration in the control group are marked with C and metabolites with higher relative concentration in the typhoid group are marked with T.

^g^Consistantly regulated refers to metabolite consistently up or down regulated in the Bangladeshi/Senegalese validation cohort and the Bangladeshi cohort (TY2, current study) and/or the Nepali cohort (TY1, previous study).
