# Supplementary material for: Reproducible diagnostic metabolites in plasma from typhoid fever patients in Asia and Africa
Source: eLife. 2017 May 9;6:e15651. doi: 10.7554/eLife.15651 (PMC5423768; doi:10.7554/eLife.15651)
Supplement: Supplementary file 3. — DOI: http://dx.doi.org/10.7554/eLife.15651.011 [file elife-15651-supp3.docx]

# **Supplementary file 3.** Additional materials and methods

## Ethical approval

The study was conducted according to the principles expressed in the Declaration of Helsinki. The Bangladesh National Research Ethical Committee (BMRC/NREC/2010-2013/1543), the Chittagong Medical College Hospital Ethical Committee, the Oxford Tropical Research Ethics Committee (OXTREC 53-09), the Research Ethics committee of the Liverpool School of Tropical Medicine and Institute Pasteur de Dakar, Senegal, and the International Vaccine Institute, Republic of South Korea gave ethical approval for the study. Informed written or thumbprint consent was taken from the subject, their parent or caretaker for all enrolees.

## Study site, population and study design

The site and recruitment in Cambodia has been descried previously [1]. Briefly, Chittagong Medical College Hospital (CMCH) is a 1,000-bed hospital serving Chittagong and the surrounding province. Adults and children (>6 months) consecutively admitted to the adult and paediatric wards at CMCH with an axillary temperature of ≥38 °C up to 48 hours after admission and history of fever for <2 weeks were eligible for the study.

This study was designed to compare blood culture alongside PCR amplification, rapid serological diagnostics tests and other approaches for the diagnosis of acute typhoid fever [1]. Blood and urine were collected from patients and controls at the time of admission to the study. Blood was collected in EDTA tubes and separated into cells and plasma on the day of collection before storage at -20^o^C on site at CMCH. Urine was collected in universal containers and stored at -20^o^C without preservatives. Here the gold standard for typhoid fever diagnosis was blood culture (5**–**12 mL for adults and 1**–**12 mL) using Bact/Alert-FA and PF blood culture bottles, bottles were incubated in the Bact/Alert automated system (Biomerieux, Marcy l’Etoile, France) for five days. The patient demographics and diagnostic testing results for this study are reported elsewhere. For the purposes of this study we randomly selected 10 patients that were culture positive for typhoid fever, 9 patients that were clinically suspected to have typhoid fever, and 11 patients with non-typhoid febrile disease; data from these patients are shown in Table 1. Urine and plasma samples from these individuals were shipped to Umeå University, Umeå, Sweden on dry ice and stored at -80^o^C until preparation for metabolomic profiling.

As a validation cohort 54 plasma samples from Bangladesh (n=21) from the above population and Senegal (n=33) were selected including samples from 14 patients with confirmed typhoid fever (Bangladesh n=9, Senegal n=5), 15 with confirmed malaria (Bangladesh n=1, Senegal n=14) and 25 febrile controls with infection caused by other bacteria/pathogens (Bangladesh n=11, Senegal n=14) (Supplementary file 5). The study setting in Senegal was Pikine, a semi-urban setting with a total population of 342,178 (2012) located in the eastern region of Senegal’s capital Dakar, was selected for passive surveillance of acute febrile patients of all ages and both sexes [2]. During December 2011 to April 2013, a total of 1,058 patients that met the inclusion criteria (tympanic temperature of ≥38.0°C or axillary temperature of ≥37.5°C; history of fever <72 hours; residing in the catchment area) consented to participate and were recruited at primary and secondary healthcare facilities [3]. A single EDTA blood sample (adults: 5-10 mL; children: 1-3 mL) was taken from each participant, which was inoculated into an aerobic blood culture bottle and incubated in an automated blood culture system. Specimens that were detected positive by blood culture were investigated further using standard microbiological techniques, and identification by biochemical reactions and agglutination tests, including antimicrobial susceptibility testing by agar disc diffusion. Plasma was stored at -80oC until shipped to Umea for metabolomic analyses.

## Sample preparation for metabolomics analysis

The plasma protocol for metabolomics at the Swedish Metabolomics Centre (SMC)[2] was used for extraction and derivatization of the plasma samples and described previously [4]. 10 quality control (QC) samples were prepared by pooling 170 µl aliquots of all plasma samples. The urine samples were prepared using a modified version of a previously described protocol [5]. Urine samples were thawed at room temperature and stored on ice; 100µl was transferred to microcentrifuge tubes (Sarstedt Ref: 72.690) for metabolite profiling. Samples were centrifuged for 10 minutes at 14,000 rpm at 4^o^C (5417R, Eppendorf, Hamburg, Germany) before 50µl of supernatant was transferred to LC vials. 100µl of water containing two isotopically labeled internal standards (Phenylalanine (U-^13^C_9_) and Caffeine (trimethyl-^13^C_3_), 0.9375 ng/µl) was added to all samples followed by 5 minutes of shaking. QC samples were prepared in a dilution series with five concentrations and two samples/concentration.

The Bangladeshi and Senegalese validation plasma samples were extracted and derivatized for GC-TOFMS analysis in the same way as the original Bangladeshi plasma samples were prepared for GCxGC-TOFMS analysis. Due to sample availability the sample volume: extraction mix volumes used were; 50:450, 35:315 and 30:270 µl. Pooled quantities of samples from a meningitis cohort were used as quality control samples (7 QC samples).

***GCxGC-TOFMS analysis of plasma samples***

Patient samples were matched broadly according to age and gender to achieve sets of three samples (*S*. Typhi culture positive, *S*. Typhi culture negative and other febrile disease). GCxGC-TOFMS analysis was performed as described previously [6], with minor modifications. The MS transfer line temperature was 325^o^C (previously 300^o^C) and the detector voltage was 1,700V (previously 1,780 V). A QC sample was run after every sixth sample in the run order and in addition blank samples (containing milli-Q water and extraction mix with internal standards only) and n-alkane series (C8-C40), for calculation of first dimension retention indexes, were included in the run.

***UHPLC-Q-TOFMS analysis of urine samples***

Extracted urine samples were analyzed on an Agilent 1290 Infinity UHPLC system (Agilent Technologies, Palo Alto, GA, USA) coupled to an Agilent 6550 iFunnel Q-TOF tandem mass spectrometer with Dual Agilent Jet Stream Electrospray Ionization. 2µl of sample was injected onto an ACQUITY UPLC HSS T3, 1.8µm, 2.1 x 50 mm column (Waters Corp, Milford, MA, USA) held at 40^o^C. Elution was performed with solvent A (water + 0.1 % formic acid) and B (acetonitrile/ isopropanol 75/25 + 0.1 % formic acid). The following gradient was used: 0-2 min, 0.1-10 % B; 2-7 min, 10-99 % B; 7-9 min 99 % B; 9-10.8 min, 0.1 % B, with a flow rate of 0.5 ml/min (with an increase to 0.8 ml/min from 9.8-10.7 min). All samples were first analysed in positive ionization mode followed by analysis in negative ionization mode. The ion source parameters were: capillary voltage: 4,000 V, gas temperature: 150^o^C, gas flow rate 16 l/min, nebulizer pressure: 35 psig, sheath gas temperature: 350^o^C, sheath gas flow rate 11 l/min, nozzle voltage: 300 V, fragmentor voltage: 380 V. The mass range was 70-1700 m/z and 4 spectra/s were collected (all in centroid mode) and the reference masses used were 121.0509 and 922.0098 m/z for positive mode and 119.0363 and 966.0007 m/z for negative mode. All samples were analysed in MS mode but in addition one QC sample was run in auto MS/MS mode at three different collision energies (10, 20 and 40 eV) where the mass range was 100-1700 m/z at a rate of 6 spectra/s. During the analysis the samples were kept at 4 ^o^C. Two QC samples (one with “low” concentration followed by one with “high” concentration, with blank samples (only containing water and internal standards) before and after the QC samples) were run between every sixth sample in the run order. Internal quality (IQ) samples were run at the beginning, between the polarity modes and at the end of the run to keep track of instrument quality.

***GC-TOFMS analysis of validation samples***

GC/MS analysis was carried out as described before with some minor changes [4]. 1 μl of each sample was injected splitless, by a CTC Combi Pal autosampler (CTC Analytics AG, Zwingen, Switzerland), into an Agilent 6890 (Agilent, Atlanta, GA, USA) gas chromatograph. A fused silica capillary column (10 m x 0.18 mm x 0.18 µm i.d.) chemically bounded to a 0.18 μm DB5-MS stationary phase column (J&W Scientific, Folsom, CA, USA) was used. The injector temperature was 270^◦^C and the purge flow started after a 60 sec delay with a rate of 20 ml/min. The helium gas flow rate through the column was 1 ml/min. Temperature programming started at an initial column temperature of 70^◦^C (with a 2 min holding time) followed by a temperature increase of 40^◦^C/min until a final temperature of 320^◦^C was reached (with a 2 min holding time). The chromatographic effluent was then directed into the ion source (200^◦^C) of a Pegasus III-TOF-MS (Leco Corp., St Joseph, MI, USA) mass spectrometer via a 250^◦^C transfer line. A 70 eV electron beam was used to ionize the molecules (with a 2.0 mA current). After a solvent delay of 150 sec mass spectra were recorded with a mass range of 50-800 m/z at a rate of 30 spectra/sec with an acceleration voltage of 1920 V. The total analysis time for each sample was around 15 min (including cooling time for the oven). As for the GCxGC-TOFMS analysis QC samples (here run in the beginning, after every tenth sample and in the end), blanks and n-alkane series were included in the run. The samples were run in a randomized order according to diagnosis and country of origin.

## Data processing and metabolite identification

For the GCxGC-TOFMS plasma metabolite data, files from the GCxGC-TOFMS instrument were converted into NetCDF format (using ChromaTOF, v4.50, Leco Corp., St Joseph, MI, USA) and processed using an in-house MATLAB script (MATLAB R2014b, Mathworks, Natick, MA, USA) using hierarchical multivariate curve resolution (HMCR) methodology, previously developed for one-dimensional GC/MS data [5] but here applied to two-dimensional data. The hierarchical multivariate curve resolution resolves a chromatographic profile (for relative quantification) and a mass spectral profile (for identification) for metabolites in all samples simultaneously. Identification of the metabolites was performed by mass spectral library searches against in-house libraries generated at SMC together with publicly available libraries from US National Institute of Science and Technology (NIST) and the Max Planck Institute in Golm (http://csbdb.mpimp-golm.mpg.de/csbdb/gmd/ gmd.html), using NIST MS Search 2.0 software. In addition, a library containing the peaks from our previous typhoid infection metabolomics study [5] was searched against to further validate putative identities as well as finding unidentified metabolites in common between the two studies. Peaks with comparable retention indexes and mass spectra or peaks from metabolites with different degree of derivatization (different number of TMS groups) were summed together and compared to the individual peaks in a PCA model to make decision about inclusion. Peaks were excluded from further analysis if a silyl artefact, an internal standard (investigated separately), were part of a summed peak, had few mass fragments in spectra or having a high correlation with the analytical run order (Pearson correlation coefficient >|0.5|). Two different methods were tested to normalize the data; using internal standards or the total abundance of the next QC sample in the analytical run order. Notably, no significant difference was observed between normalized and non-normalized data, therefore data was kept non-normalized throughout the analysis.

For the UHPLC-Q-TOFMS urine metabolite data, files in .d format were imported to Mass Hunter Qualitative Analysis (B.06.00, Agilent) where the Find Compound by Molecular Feature (MFE) algorithm was used for peak picking (a value of 2-3 times the baseline was used as threshold for the peak filtering, the number of charge states was limited to one and two or more ions allowed) (to automate the MFE extraction for all samples the Mass Hunter DA Reprocessor (B.04 software, Agilent). The resulting CEF files containing the extracted molecular features were imported into Mass Profiler Professional (MPP) (B.12.05, Agilent) for peak filtering and compound alignment (the minimum absolute abundance was set to 5,000 counts, the minimum number of ions was set to 2, multiple charge states were forbidden, compound alignment was performed with a RT window of 0 % + 0.15 min and a mass window of 50 ppm + 2.0 mDa and finally the peak should be present in at least 67 % in at least one of the three samples groups (other febrile disease, *S*. Typhi culture positive and *S*. Typhi culture negative)). The peak list from MPP was then exported for recursion using the Find by Formula (FBF) algorithm in Mass Hunter Qualitative Analysis (mass and retention time values were used for matching, the match tolerance was 50 ppm for masses and 0.350 minutes for retention times, the EIC Peak Filter was set to a height of 1,000 counts). The resulting CEF files were imported to MPP for final analysis (no filtering was performed and the alignment of the FBF features was performed with wide RT and mass windows). The internal standards were quantified using Mass Hunter Quantitative Analysis (B.05.02). The initial numbers of peaks were reduced in a number of filtering steps; a peak had to be detected in ≥ 7 samples in at least one of the sample classes and also in ≥ 7 of the QC samples. Again peaks with a high degree of correlation with the analytical run order were excluded (Pearson correlation coefficient for analytical run order >|0.5|). In addition, the QC dilution series was used to remove peaks not increasing in measured levels with increasing concentration of sample in the QC series (Pearson correlation coefficient for QC dilution series ≤0.75).

For the GC-TOFMS plasma validation data files were converted into NetCDF format and processed using an in-house MATLAB script using a targeted approach. The data processing included sample alignment (with multiple internal standards), retention index calculation, peak detection, mass spectra deconvolution, mass spectra library search (targeting an in-house metabolite library) and calculation of peak area. The resulting peaks from the processing were manually investigated and further mass spectral library search were performed in NIST MS Search 2.0 (the spectra were also compared to spectra from the original plasma GCxGC-TOFMS data and from the previous typhoid infection metabolomics study). Similar to the GCxGC-TOFMS Bangladeshi plasma data peaks that had comparable retention indexes, mass spectra and position in PCA loadings were summarized. Analytical run order correlation was calculated but no peak had a correlation that exceeded the set threshold (Pearson correlation coefficient >|0.5|).

## Pattern recognition

The measurement of several hundred metabolites results in highly multi-dimensional data requiring data analysis strategies able to deal with multiple covariates. Principal components analysis (PCA) [8] was used to obtain an overview of the variation in the metabolomic data by means of unsupervised modelling, to visualize general trends and detect possible outliers. This was followed by supervised modelling by means of orthogonal partial least squares-discriminant analysis (OPLS-DA) [9], a multivariate discriminant analysis method where pre-defined classes are compared with the goal of finding metabolite patterns that discriminates between the sample groups. OPLS [10] is an extension of partial least squares (PLS) [11], where the variation in the measured variables, X (here the semi-quantified metabolite concentrations) is divided into one part related to the response of interest, Y (here class information regarding presence of *S*. Typhi infection) (predictive variation) and one part unrelated to the response of interest (orthogonal variation) for facilitated interpretation. OPLS-DA models were obtained for comparing *S*. Typhi culture positive vs. control for both plasma and urine. The obtained OPLS-DA models for comparing *S*. Typhi culture positive sample and other febrile disease samples were then used to predict the class belonging of the *S.* Typhi culture negative samples. For the validation data, a 3-class OPLS-DA model was calculated for the separation between typhoid malaria and other bacteria/pathogens. Furthermore, separate OPLS-DA models were calculated for the separation between typhoid and all controls (malaria and other bacteria/pathogens as one class) as well as between typhoid and malaria. For all models samples from both Bangladesh and Senegal were included. All OPLS-DA models were validated by full cross-validation and described by the number of principal components, the amount of variation in X explained by the model (R^2^X), the amount of variation in Y explained by the model (R^2^Y), the amount of variation in Y predicted by the model (Q^2^) and probability values for the degree of significance of the class separation of interest (CV-ANOVA) [12]. Centred and scaled data (unit variance) was used throughout the modelling. Model covariance loadings (w*) were used to determine significantly altered metabolites or metabolite patterns (w*> | ±SD|). In addition, univariate p-values were used to highlight metabolites with univariate significance (p ≤ 0.05) (due to mixed outcome of the Shapiro-Wilk test for normality p-values were calculated using a two-tailed Student´s t-test as well as the Mann Whitney U-test). All pattern recognition modelling was performed in SIMCA (version 14, Umetrics, Umeå, Sweden). SIMCA in combination with Adobe Illustrator CS5 (15.0.0, Adobe Systems Inc., San Jose, CA, USA) was used to create model figures.

***Comparison of metabolites between studies***

To validate our results from the previous typhoid infection metabolomics study, in a Nepali cohort, [5] the detected metabolite pattern from the OPLS-DA models of culture positive typhoid infection and controls in the Bangladeshi cohort were compared to the corresponding pattern in the Nepali cohort to detect metabolites present and significant in both studies. The metabolites were compared based on identity and mass spectra and thus both putatively identified and unidentified metabolites could be included as common between studies. For metabolites common between the studies the degree of multivariate significance and direction of change in the models were compared. Two different multivariate significance levels for the current study were used in the comparison, w* > |0.03| to represent the same significance level as used in the previous study and w* > | ±SD| approximately w* > |0.06|) to represent a stricter selection. Further comparisons of metabolite patterns were made between the validation data and the Bangladeshi and Nepali cohorts. Detected metabolite patterns from the OPLS-DA model separating typhoid and controls for the Bangladeshi and Senegalese validation cohort were compared to the corresponding patterns in the Bangladeshi cohort in the current study and furthermore in the Nepali cohort from the previous study. Again metabolites were compared based on identity, mass spectra, degree of multivariate significance and direction of change. Two multivariate significance criteria were used as for the original samples (w* > |0.03| and w* > | ±SD|).

**Supplementary references**

1. Maude RR, de Jong HK, Wijedoru L, Fukushima M, Ghose A, Samad R, et al. The diagnostic accuracy of three rapid diagnostic tests for typhoid fever at Chittagong Medical College Hospital, Chittagong, Bangladesh. Trop Med Int Health. 2015;20: 1376–84. doi:10.1111/tmi.12559

2. v Kalckreuth V. et al. The Typhoid Fever Surveillance in Africa Program (TSAP): Clinical, Diagnostic, and Epidemiological Methodologies. Clin Infect Dis, v. 62 Suppl 1, p. S9-S16, Mar 2016.

3. Marks F. et al. Incidence of invasive salmonella disease in sub-Saharan Africa: a multicentre population-based surveillance study. Lancet Glob Health, v. 5, n. 3, p. e310-e323, Mar 2017.

4. A J, Trygg J, Gullberg J, Johansson AI, Jonsson P, Antti H, et al. Extraction and GC/MS Analysis of the Human Blood Plasma Metabolome. Anal Chem. 2005;77: 8086–8094. doi:10.1021/ac051211v

5. Näsström E, Thieu Nga TV, Dongol S, Karkey A, Voong Vinh P, Ha Thanh T, et al. Salmonella Typhi and Salmonella Paratyphi A elaborate distinct systemic metabolite signatures during enteric fever. Elife. 2014; e03100. doi:10.7554/eLife.03100

6. Want EJ, Wilson ID, Gika H, Theodoridis G, Plumb RS, Shockcor J, et al. Global metabolic profiling procedures for urine using UPLC–MS. Nat Protoc. 2010;5: 1005–1018. doi:10.1038/nprot.2010.50

7. Jonsson P, Johansson AI, Gullberg J, Trygg J, A J, Grung B, et al. High-Throughput Data Analysis for Detecting and Identifying Differences between Samples in GC/MS-Based Metabolomic Analyses. Anal Chem. 2005;77: 5635–5642. doi:10.1021/ac050601e

8. Wold S, Esbensen K, Geladi P. Principal component analysis. Chemom Intell Lab Syst. 1987;2: 37–52. doi:10.1016/0169-7439(87)80084-9

9. Bylesjö M, Rantalainen M, Cloarec O, Nicholson JK, Holmes E, Trygg J. OPLS discriminant analysis: combining the strengths of PLS-DA and SIMCA classification. J Chemom. 2006;20: 341–351. doi:10.1002/cem.1006

10. Trygg J, Wold S. Orthogonal projections to latent structures (O-PLS). J Chemom. 2002;16: 119–128. doi:10.1002/cem.695

11. Wold S, Sjöström M, Eriksson L. PLS-regression: a basic tool of chemometrics. Chemom Intell Lab Syst. 2001;58: 109–130. doi:10.1016/S0169-7439(01)00155-1

12. Eriksson L, Trygg J, Wold S. CV-ANOVA for significance testing of PLS and OPLS® models. J Chemom. 2008;22: 594–600. doi:10.1002/cem.1187
